# Hydrogen-rich water alleviates the toxicities of different stresses to mycelial growth in *Hypsizygus marmoreus*

**DOI:** 10.1186/s13568-017-0406-1

**Published:** 2017-05-30

**Authors:** Jinjing Zhang, Haibo Hao, Mingjie Chen, Hong Wang, Zhiyong Feng, Hui Chen

**Affiliations:** 1 National Research Center for Edible Fungi Biotechnology and Engineering, Key Laboratory of Applied Mycological Resources and Utilization, Ministry of Agriculture, Shanghai Key Laboratory of Agricultural Genetics and Breeding, Institute of Edible Fungi, Shanghai Academy of Agricultural Sciences, No. 1000, Jinqi Road, Fengxian District, Shanghai, 201403 China; 2 College of Life Science, Nanjing Agricultural University, No.1, Weigang Road, Xuanwu District, Nanjing, 210095 China

**Keywords:** *Hypsizygus marmoreus*, Hydrogen gas, Oxidative stress, Mycelial growth

## Abstract

In plants, hydrogen gas (H_2_) enhances tolerance to several abiotic stresses, including salinity and heavy metals. However, the effect of H_2_ on fungal growth under different stresses remains largely unclear. In this study, hydrogen-rich water (HRW) was employed to characterize physiological roles and molecular mechanisms of H_2_ in the alleviation of three different stresses in basidiomycete *Hypsizygus marmoreus*. Our results showed that HRW treatment, of which the H_2_ concentration was 0.8 mM, significantly reduced the toxicities of CdCl_2_, NaCl and H_2_O_2_, leading to significantly improved mycelial growth and biomass. These beneficial effects could be attributed to a significantly decreased formation of malondialdehyde (MDA). Besides, HRW treatment significantly increased the activities of antioxidants (SOD, CAT and GR) as well as the gene expressions of these antioxidants (SOD, CAT, and GR) at the mRNA level. In vivo detection of reactive oxygen species (ROS), including H_2_O_2_ and O_2_^−^, as well as lipid peroxidation provided further evidence that HRW could significantly improve tolerances of CdCl_2_, NaCl and H_2_O_2_. Furthermore, pyruvate kinase was activated in the mycelia treated with HRW, along with its induced gene expression, suggesting that HRW treatment enhanced the glucose metabolism. Taken together, our findings suggested that the usage of HRW could be an effective approach for contaminant detoxification in *H. marmoreus*, which was similar with the effects of HRW in plants, and such effects could be also beneficial in entire agricultural system.

**Electronic supplementary material:**

The online version of this article (doi:10.1186/s13568-017-0406-1) contains supplementary material, which is available to authorized users.

## Introduction

*Hypsizygus marmoreus* (Peck.) Bigelow (Tricholomataceae), also known as bunashimeji and hon-shimeji, has been successfully and commercially cultivated in East Asia (Akavia et al. 2006). In China, *H. marmoreus* has become increasingly popular due to its mild, sweet, nutty flavor and crunchy texture as well as some physiologically beneficial components. Nowadays, the substrates used for culturing mushrooms from plants, such as sawdust, corncob and wheat bran, are usually contaminated. Contaminations will severely inhibit mushroom growth, which may reduce the fruit body production. If the contaminations, such as heavy metals, are accumulated in crop plants, they will also accumulate in the fruit body of mushroom, posing a severe threat to human health through food chains (Järup and Åkesson 2009; Podazza et al. 2012).

In fungi, the oxidative stress, at least in part, is caused by stimulated generation of reactive oxygen species (ROS), which is able to modify the antioxidant defense and elicit oxidative stress (Rodríguez-Serrano et al. 2006, 2009; Schützendübel et al. 2001). ROS, including *OH, O_2_^−^ and H_2_O_2_, if uncontrolled, can cause oxidative damage to macromolecules, such as lipids, thus leading to lipid peroxidation and cell death (Bailly 2004). The enzymatic system, including superoxide dismutase (SOD), catalase (CAT), glutathione peroxidase (GPX) and guaiacol peroxidase (POD), can scavenge the ROS and enhance the fungal growth.

Recently research has found that hydrogen gas (H_2_) is a potentially ‘novel’ antioxidant in plants and animals. As the most abundant chemical element in the universe, H_2_ is a colorless, odorless, tasteless and highly combustible diatomic gas that has been known for many years (Huang et al. 2010). However, direct use of H_2_ is dangerous and flammable (Xie et al. 2012). Therefore, most of researchers use the hydrogen-rich water (HRW) to perform experiments, which is safe, cost-effective and commercially available. In plants and animals, HRW has been widely used as an antioxidant (Ohta 2012; Xie et al. 2012).

In animals, HRW, as an antioxidant, has beneficial effects in preventive and therapeutic applications, and such effects have been reported in 38 diseases and physiological states, including Parkinson, atherosclerosis, glaucoma and hepatic ischemia disease (Ohta 2012). In plants, HRW has been also used to illustrate that H_2_ can act as a novel beneficial gaseous molecule in plant adaptive responses (Cui et al. 2013; Jin et al. 2013; Wu et al. 2015a; Xie et al. 2012). In Chinese cabbage, HRW improves CdCl_2_ tolerance by reducing CdCl_2_ uptake and increasing antioxidant defense (Wu et al. 2015b). In Arabidopsis, HRW can enhance its salt tolerance by increasing antioxidant defense and significantly counteracting the NaCl-induced ROS overproduction and lipid peroxidation (Xie et al. 2012). Under high light stress, HRW decreases the levels of O_2_^−^ and H_2_O_2_ and elevates the activities of antioxidants, including SOD, CAT, APX and GR (Zhang et al. 2015a). Besides, HRW can activate α/β-amylase activity, thus accelerating the formation of reducing sugar and total soluble sugar in rice (Xu et al. 2013). However, there is not report about the effects of HRW on the fungal growth in stressful environment.

In the present study, we evaluated the effects of HRW on the mycelial growth under three types of stresses (CdCl_2_, NaCl and H_2_O_2_) and found that HRW treatment enhanced the mycelial growth and increased the mycelial biomass. In addition, HRW could enhance the antioxidant activities, decreased the ROS level and alleviated the lipid peroxidation in mycelia of *H. marmoreus*. Moreover, HRW also activated the pyruvate kinase (PK) in the mycelia. These results suggested a positive role of HRW in reducing pollutant residues for mushroom safety.

## Materials and methods

### Fungal materials, growth conditions, experimental design and growth analysis

*Hypsizygus marmoreus* samples were obtained from the China General Microbiological Culture Collection Center (Beijing) (No. CGMCC5.01974). First, the mycelium of *H. marmoreus* was cultured on PDA medium at 25 °C for 2 weeks, and then it was transferred onto PDB and PDA medium containing CdCl_2_, NaCl or H_2_O_2_ of different concentrations. The mycelial biomass was determined on PDB medium, and the mycelial growth was examined on PDA medium under various stresses. The 50% inhibition concentrations of CdCl_2_, NaCl and H_2_O_2_ were also evaluated accordingly.

Besides, the mycelia were transferred into solutions containing 0 or 50% inhibition concentration of CdCl_2_, NaCl and H_2_O_2_ and then incubated at 25 °C for 24 h. Subsequently, the mycelia were transferred into HRW for 5 days, and the HRW was replaced every 12 h. Mycelia without HRW treatment were used as the control (H_2_O). These above-mentioned treatments could be described as follows: (1) H_2_O → H_2_O, H_2_O → HRW, CdCl_2_ → H_2_O, CdCl_2_ → HRW, H_2_O_2_ → H_2_O, H_2_O_2_ → HRW, NaCl → H_2_O, NaCl → HRW. In addition, the mycelial growth was determined after the HRW treatment for 5 days. Meanwhile, the mycelial biomass was determined on PDB medium in the similar experimental treatment. Before being added to the medium, CdCl_2_, NaCl or H_2_O_2_ solutions were sterilized by filtration through a 0.22-μm membrane. Co-treatment with HRW was applied on the 8th day, and 0 or 50% inhibition concentration of CdCl_2_, NaCl or H_2_O_2_ were added on the 9th day and then incubated at 25 °C for 24 h respectively. Mycelia were harvested and washed with a large amount of distilled water and then dried at 60 °C to a constant biomass. The Growth tests were performed in triplicate. Ruler (0.1 cm) and electronic balance (0.0001 g) were used in measurements of mycelial growth and biomass after various treatments.

## Determination of H_2_ concentrations

HRW was kindly supplied by Beijing Hydrovita Beverage Co., Ltd. (Beijing, China) and the H_2_ concentration in the freshly HRW was 1.0 mM at a hermetical canister. While the canister was opened, the H_2_ concentration was 0.8 mM in 30 min. The H_2_ concentration was maintained at a relative constant level in 25 °C for at least 12 h. The H_2_ concentration was analyzed by using gas chromatography (GC). The chromatographic system (GC 7890, Agilent) was according the method described by Wu et al. (2015a) which was equipped with thermal conductivity detector (TCD). The working conditions were optimized as TCD detector temperature at 100 °C, 5 Å molecular sieves as fixed phase, column temperature at 150 °C, oven temperature at 60 °C. Nitrogen gas was used as carrier gas and air pressure 0.2 MPa.

### Histochemical detection of ROS

H_2_O_2_ or O_2_^−^ level was measured by 3, 3, 9-diaminobenzidine (DAB) or nitroblue tetrazolium (NBT) staining, respectively. First, the mycelia were cultured on PDB medium at 25 °C 150 rpm for 10 days, and then the mycelia became mycelial pellet. Subsequently, different stresses were added into the PDB medium to make the PDB medium contained the 50 μM CdCl_2_, 1% NaCl or 2 mM H_2_O_2_ respectively. After the mycelia were cultured for 24 h, the mycelial pellets were collected and transferred into ddH_2_O or HRW for 48 h. Finally, the mycelial pellets were immersed in freshly prepared DAB solution (0.1% w/v, pH 3.8), vacuum-infiltrated, and then incubated at 25 °C for 2 h in darkness. Alternatively, the mycelial pellets were immersed in NBT solution consisting of 10 mM potassium phosphate (pH 7.8) and 10 mM NaN_3_, vacuum-infiltrated, and then incubated at 25 °C for 1 h in darkness. After extensive wash, all the decolorized mycelial pellets were examined on a color film (Powershot G16; Canon Photo Film, Tokyo, Japan).

### Detection of H_2_O_2_ and MDA concentrations

The organism produces oxygen free radicals through enzyme system and non-enzyme system. H_2_O_2_ is the product of the enzyme system. Fresh mycelia (1.0 g) were homogenized in a mortar with 10 mL physiological saline on ice. Then, the obtained 10% homogenate was centrifuged at 2500*g* for 10 min. The supernatant was used to detect the concentration of H_2_O_2_, which was analyzed using Hydrogen Peroxide assay kit (NJJCBio. Ltd., China). The absorbance of the supernatant was read at a wavelength of 405 nm, and ddH_2_O was used as the blank.

H_2_O_2_ can induce the lipid peroxidation by attacking poly unsaturated fatty acid in the biological membrane and then form lipoid peroxide, injuring the cells and tissue. The malondialdehyde (MDA) was used as an indicator of oxidative stress in non-enzyme system. The supernatant of 10% homogenate was also used to detect the concentration of MDA, which was determined by MDA assay kit (TBA method) (NJJCBio. Ltd., China). The absorbance of the supernatant was read at a wavelength of 532 nm, and ddH_2_O was used as the blank.

### Antioxidant activity assays

Fresh mycelia (1.0 g) were homogenized in 9.0 mL of 0.1 M phosphate buffer (PH 7.0) on ice. Then, the 10% homogenate was centrifuged at 4000*g* for 10 min at 4 °C, and the supernatant was used for assays of superoxide dismutase (SOD), catalase (CAT) and glutathione reductase (GR) activities. The CAT activity was detected according to the instruction of Catalase (CAT) assay kit (Visible light) (Nanjing Jiancheng Bioengineering Institute, Nanjing, China). One unit of CAT was defined as the mount of enzyme for decomposing 1 μmol H_2_O_2_ monitored at 405 nm. The SOD activity was detected as description by Total Superoxide Dismutase (T-SOD) assay kit (Hydroxylamine method) (Nanjing Jiancheng Bioengineering Institute, Nanjing, China). One unit of SOD activity was defined as the amount of SOD while inhibition of SOD was up to 50% per gram tissue in 1 mL reacted solution and the SOD activity was monitored at 550 nm. The GR activity was detected as description by glutathione reductases assay kit (Nanjing Jiancheng Bioengineering Institute, Nanjing, China) monitored at 340 nm.

### Total protein detection

The protein concentration of mycelia used for calculating was detected as described by Total protein quantitative assay kit (Coomassie Brilliant Blue) (Nanjing Jiancheng Bioengineering Institute, Nanjing, China) monitored at 595 nm. The standard protein concentration used in this assay kit was 0.563 g/L and the color solution was the coomassie brilliant solution.

### Determination of CdCl_2_ and NaCl content in mycelia

The concentrations of CdCl_2_ were determined using an atomic absorption spectrophotometer (180-80 Hitachi, Tokyo, Japan) as described by Liu et al. (2008). The concentration of NaCl element was measured by an Inductively Coupled Plasma–Optical Emission Spectrometer (ICP–OES, Perkin Elmer Optima 2100DV) by using the total sodium content kit (Comin Biotechnology, Suzhou, China).

### Real-time quantitative RT-PCR analysis

Total RNA was isolated from fresh mycelia using Trizol reagent (Takara, Dalian, China) according to the manufacturer’s instructions. The RNA purity was verified based on the ratio (>2.0) of 260/280 nm using the gDNA assay kit (Takara, Dalian, China). Subsequently, 20 μL purified RNA was reversely transcribed into cDNA in a 40-μL reaction system according to the manufacturer’s instructions. Real-time quantitative PCR reactions were performed as described by Zhang et al. (2014, 2015a, b) using SYBR (Takara, Dalian, China). Additional file 1: Table S1 lists the primer sequences and accession numbers of the target genes, and 18S ribosomal RNA was selected as the housekeeping gene in the present study. Each experiment was performed in triplicate. The relative gene expression was analyzed using the 2^−ΔΔCt^ method described by Livak and Schmittgen (2001).

### Data presentation and statistical analysis

Values are shown as the mean ± SD of three independent experiments with three replicates each. Differences among treatments were analyzed by one-way analysis of variance (ANOVA) combined with Duncan’s multiple range test at a probability of P < 0.05.

## Results

### Effects of CdCl_2_, NaCl and H_2_O_2_ on the mycelial growth

**Fig. 1:**
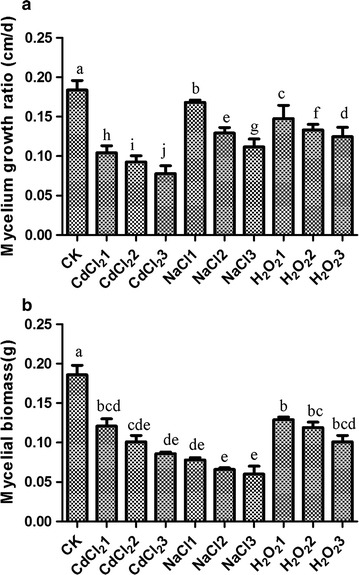
Effects of different concentrations of CdCl_2_, NaCl and H_2_O_2_ on mycelial growth and biomass of *Hypsizygus marmoreus*. Mycelia were incubated with different concentrations of CdCl_2_, NaCl and H_2_O_2_. The mycelial growth was detected on PDA medium after 10 days of incubation (**a**), and the mycelial biomass was detected on PDB medium after 10 days of incubation (**b**). Data are mean ± SE of three independent experiments. *Bars* with *different letters* are significantly different at P < 0.05 according to Duncan’s multiple range test

To detect the sensitivity of *H. marmoreus* to CdCl_2_, NaCl and H_2_O_2_, mycelia were cultured on the PDA and PDB medium with different concentrations of CdCl_2_, NaCl and H_2_O_2_. The concentrations of CdCl_2_ were 50, 100 and 150 μM, the concentrations of NaCl were 0.5, 1 and 2%, and the concentrations of H_2_O_2_ were 1, 2 and 4 mM. Figure 1 shows that the mycelial growth was markedly inhibited by the three types of stresses, exhibiting significantly decreased mycelial growth (Fig. 1a) and mycelial biomass (Fig. 1b). Therefore, 50 μM CdCl_2_, 1% Na and 2 mM H_2_O_2_ were subsequently used to investigate the role of HRW in the alleviation of inhibitory effects of the three stresses on mycelial growth.

### HRW alleviates the inhibitory effects of CdCl_2_, NaCl and H_2_O_2_ on mycelial growth

**Fig. 2:**
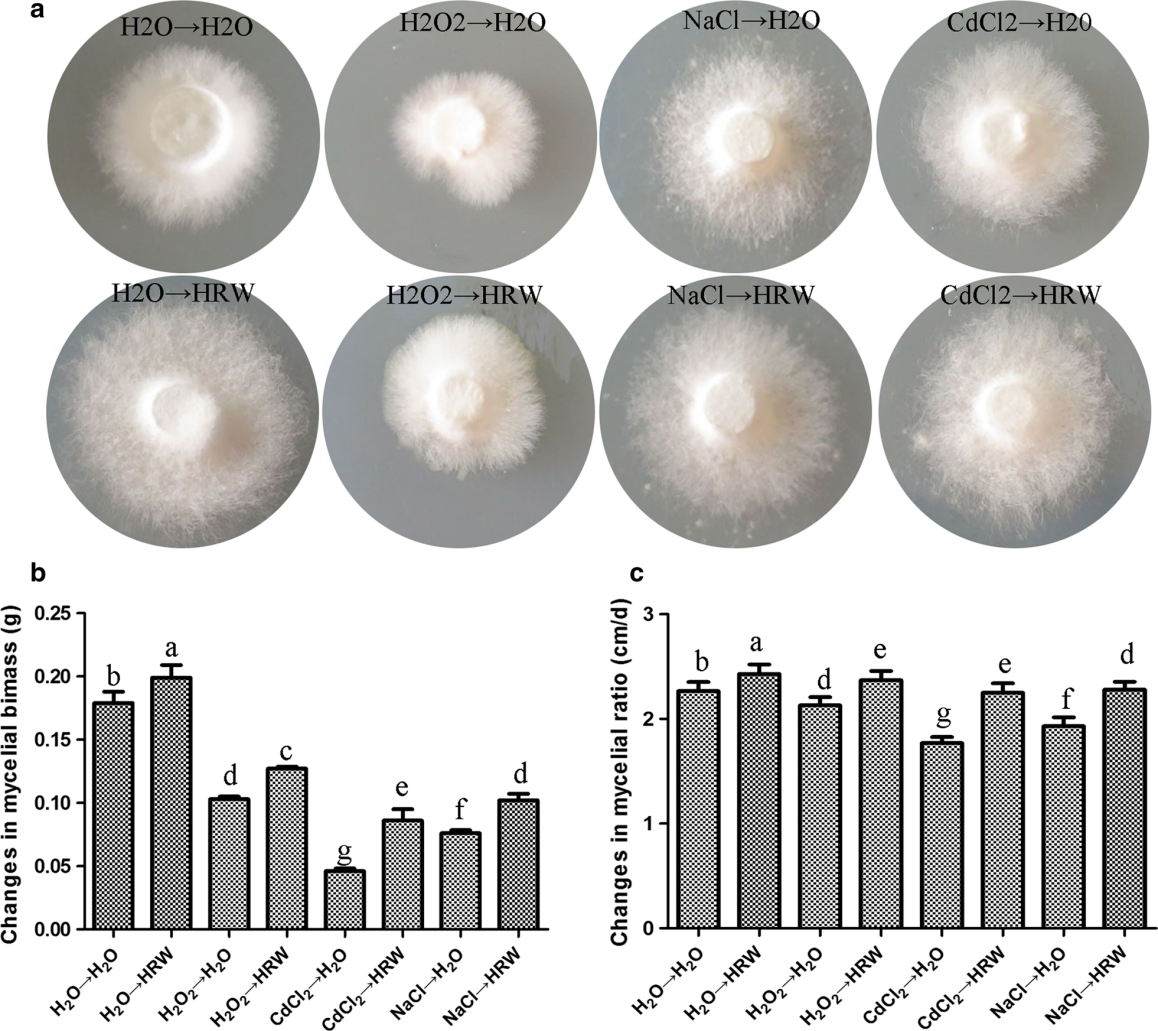
HRW treatment alleviates CdCl_2_, NaCl and H_2_O_2_ stresses. The mycelia were supplemented with 50 μM CdCl_2_, 1% NaCl or 4 mM H_2_O_2_ for 24 h, followed by incubation in ddH_2_O or HRW for 5 days on PDA or PDB medium to assess mycelial growth (**a**, **c**) and biomass (**b**). Data are mean ± SE from three independent experiments. *Bars* with *different letters* are significantly different at P < 0.05 according to Duncan’s multiple range test

To evaluate the effects of HRW on the mycelial growth under three different stresses, we placed mycelial pellets in the solutions for 24 h and then transferred them into solutions with H_2_O or HRW for 5 days. During this process, HRW was replaced every 12 h. The mycelial growth was determined after 5 days of cultivation. Figure 2a and c display that the HRW treatment could enhance the mycelial growth by alleviating the toxicities of CdCl_2_, NaCl and H_2_O_2_ to mycelia (Fig. 2a, c).

Besides, the effects of HRW on the mycelial biomass under three different stresses were assessed on PDB medium. If the three stresses were added at the early stage of cultivation, the mycelia could not grow and died finally. Therefore, after 10 days of mycelial culture, the three stresses (50 μM CdCl_2_, 1.0% NaCl or 2 mM H_2_O_2_) were respectively added into the PDB medium, and the culture was maintained for another 24 h. The stresses were then removed, and the mycelia were transferred into HRW for 5 days. Subsequently, mycelia were collected and dried at 65 °C until the biomass was stable. Figure 2b shows that the HRW treatment could significantly increase the mycelial biomass compared with the control (CK) group. Besides, the HRW treatment significantly slowed down the accumulation of CdCl_2_, H_2_O_2_ and NaCl. The CdCl_2_, NaCl and H_2_O_2_ contents in the mycelia was 20.08, 9.89 and 30.39% lower than the CdCl_2_, NaCl and H_2_O_2_-stressed alone mycelia (Table 1).

**Table 1:** Effects of HRW treatment on CdCl_2_, NaCl and H_2_O_2_ concentrations in mycelia of *H. marmoreus*

Treatments	CdCl_2_ (nmol/L)	H_2_O_2_ (mmol/g)	NaCl (mg/g)
H_2_O → H_2_O	ND^c^	62.4 ± 4.328^c^	0.101 ± 0.008^c^
H_2_O → HRW	ND^c^	60.1 ± 6.301^c^	0.098 ± 0.003^c^
CdCl_2_ → H_2_O	230.78 ± 9.876^a^	–	–
CdCl_2_ → HRW	184.62 ± 11.752^b^	–	–
NaCl → H_2_O	–	–	2.307 ± 0.506^a^
NaCl → HRW	–	–	1.606 ± 0.340^b^
H_2_O_2_ → H_2_O	–	132.18 ± 9.672^a^	–
H_2_O_2_ → HRW	–	119.11 ± 11.012^b^	–

8-day-old mycelia were pretreated with 0, 50 μm CdCl_2_, 1.0% NaCl and 2 mM H_2_O_2_ for 24 h and then were treated with or without treatment with 100% HRW. The control group was treated with water. Values are mean ± SE of three independent experiments with at least three replicates for each. Different letters within columns indicate significant differences (P < 0.05) according to Duncan’s multiple range test

### HRW enhances the antioxidant activities of mycelia

**Fig. 3:**
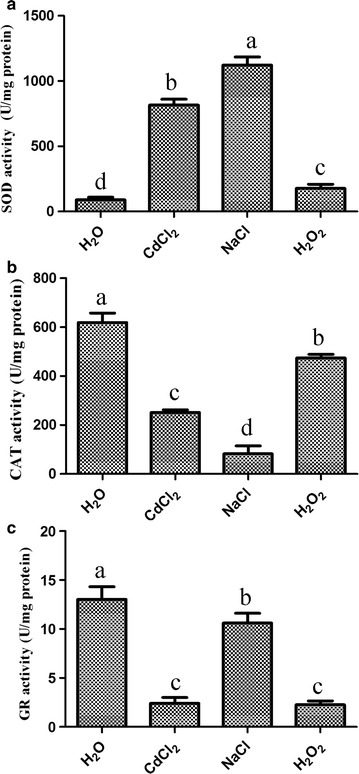
Effects of CdCl_2_, NaCl and H_2_O_2_ treatments on SOD (**a**), CAT (**b**) and GR (**c**) activities in mycelia of *Hypsizygus marmoreus*. The mycelia were cultured on PDB medium for 10 days, followed by 24 h incubation with CdCl_2_, NaCl and H_2_O_2_. Data are mean ± SE from three independent experiments. *Bars* with *different letters* are significantly different at P < 0.05 according to Duncan’s multiple range test

**Fig. 4:**
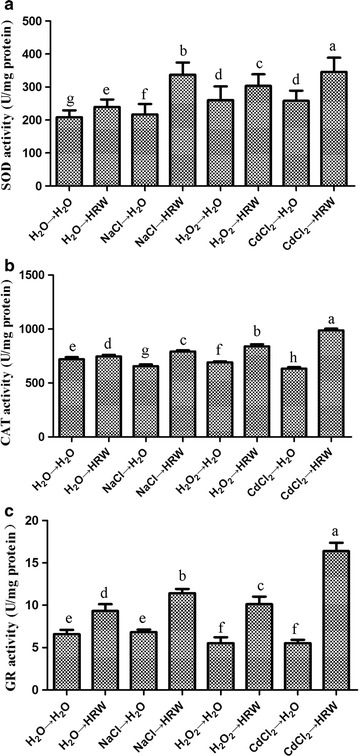
Effects of HRW treatment on SOD (**a**), CAT (**b**) and GR (**c**) activities in mycelia of *Hypsizygus marmoreus* upon CdCl_2_, NaCl and H_2_O_2_ stresses. The mycelia were pretreated with CdCl_2_, NaCl or H_2_O_2_ for 24 h, followed by 48-h incubation with or without HRW. Data are mean ± SE from three independent experiments. *Bars* with *different letters* are significantly different at P < 0.05 according to Duncan’s multiple range test

After the mycelia were cultured on PDB medium containing 50 μM CdCl_2_, 1% NaCl or 2 mM H_2_O_2_ for 24 h, the activities of several antioxidants, including CAT, SOD and GR, were determined. Figure 3 shows the sensitivity of the three antioxidants to CdCl_2_, NaCl and H_2_O_2_. The SOD activity was significantly inhibited by CdCl_2_, NaCl and H_2_O_2_. In contrast, the activities of CAT and GR were significantly induced by CdCl_2_, NaCl and H_2_O_2_. The pretreated mycelia were transferred into H_2_O or HRW for 5 days, and the mycelia were used to determine the enzyme activities of the three antioxidants. Figure 4 reveals that the enzyme activities of the three antioxidants were restored after the HRW treatment compared with the CK group, indicating that the HRW treatment induced the activities of the antioxidants. Moreover, higher CAT and GR activities were detected after the co-treatment with HRW compared with pretreatment of CdCl_2_, NaCl and H_2_O_2_ alone.

### HRW alleviates ROS homeostasis and lipid peroxidation

**Fig. 5:**
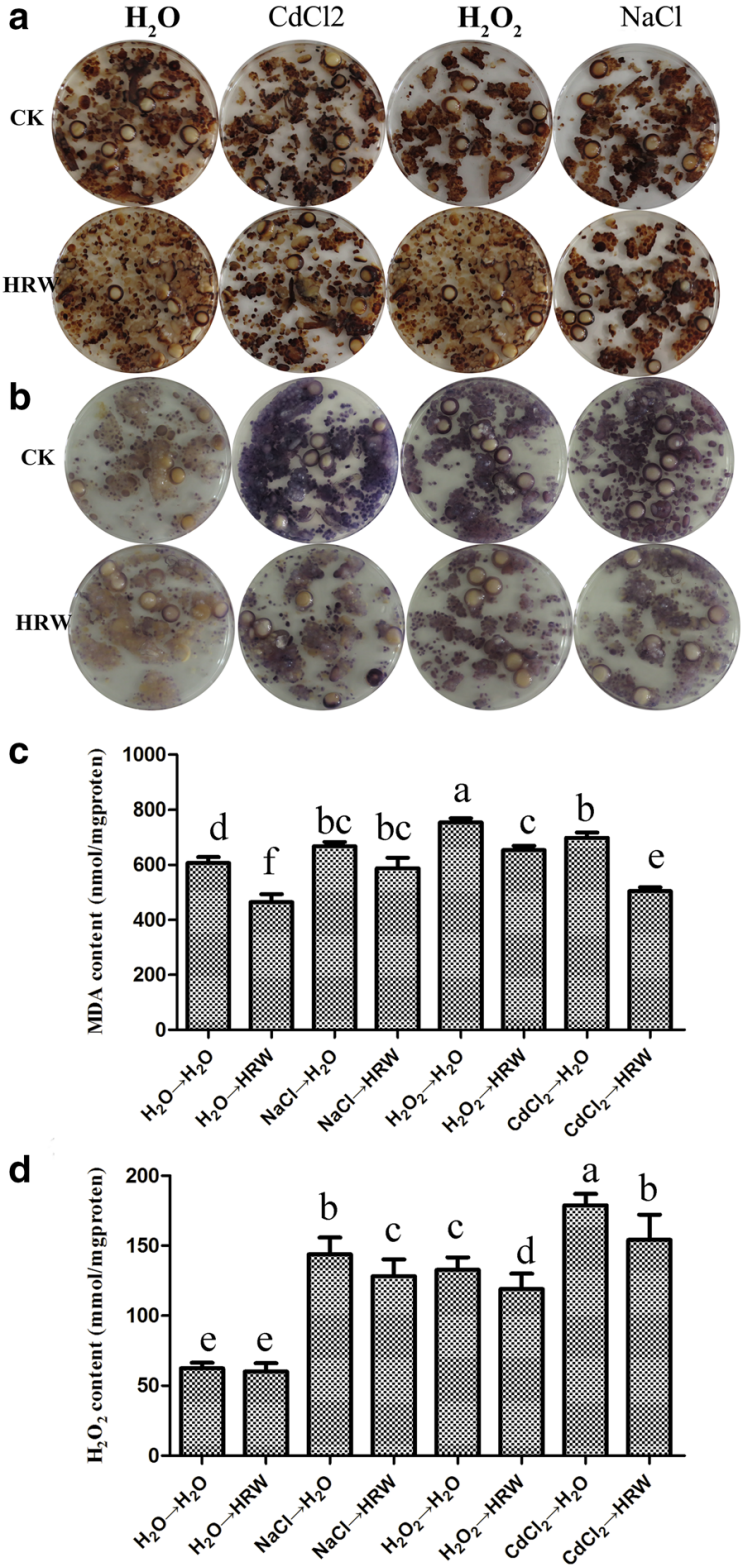
HRW protects mycelial growth against lipid peroxidation and ROS homeostasis induced by CdCl_2_, NaCl and H_2_O_2_ stresses in *Hypsizygus marmoreus*. Mycelia were pretreated with 50 μM CdCl_2_, 1% NaCl or 4 mM H_2_O_2_ for 24 h and then exposed to ddH_2_O or HRW for another 48 h. The mycelia were stained with DAB (**a**) and NBT (**b**) and 48 h after various treatments to detect H_2_O_2_ and O_2_^−^, respectively. Levels of lipid peroxidation (MDA) were measured at the indicated time points (**c**), and the levels of H_2_O_2_ were also determined (**d**). Data are mean ± SE from three independent experiments. *Bars* with *different letters* are significantly different at P < 0.05 according to Duncan’s multiple range test

The effects of HRW on the ROS production induced by CdCl_2_, NaCl and H_2_O_2_ were investigated by histochemical straining, including NBT staining for O^2−^ and DAB staining for H_2_O_2_. The mycelia were cultured on PDB medium containing CdCl_2_, NaCl and H_2_O_2_ for 24 h and then transferred into ddH_2_O or HRW for 5 days. Figure 5 exhibits that low levels of H_2_O_2_ (DAB staining) (Fig. 5a) and O_2_^−^ (NBT staining) (Fig. 5b) were detected in the HRW-treated mycelia. However, ddH_2_O-treated mycelia had higher production of H_2_O_2_ (DAB staining) and O_2_^−^ (NBT staining) compared with the HRW-treated mycelia. Besides, the levels of H_2_O_2_ were also detected using the H_2_O_2_ detection assay kit. Figure 5c shows that the levels of H_2_O_2_ in HRW-treated mycelia were lower compared with the ddH_2_O-treated mycelia. These results were consistent with the DAB staining.

In addition, the MDA formation, which is a reliable marker of lipid peroxidation and free radical generation, was examined to clarify whether the beneficial effects of HRW were related to oxidative stress. After 24-h pretreatment of CdCl_2_, NaCl and H_2_O_2_, mycelia were transferred into ddH_2_O or HRW for another 48 h. As expected, the ddH_2_O treatment caused significantly increased MDA content (Fig. 5d). However, the HRW treatment triggered a significant reduction in MDA level.

### HRW enhances the PK activity

**Fig. 6:**
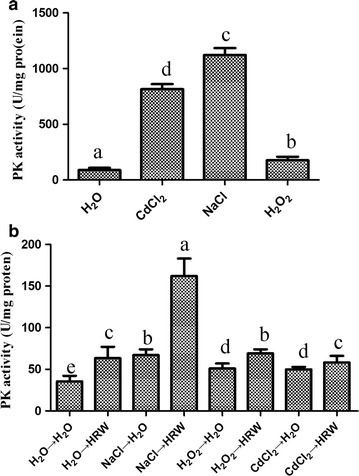
Effects of HRW treatment on PK activity in the mycelia of *Hypsizygus marmoreus*. The PK activity was determined in the mycelia pretreated with CdCl_2_, NaCl or H_2_O_2_ (**a**) and then treated with HRW for 48 h (**b**). Data are mean ± SE from three independent experiments. *Bars* with *different letters* are significantly different at P < 0.05 according to Duncan’s multiple range test

As HRW could enhance the mycelial growth by reducing the oxidative stress, we detected the effect of HRW on the activity of PK, which is an important enzyme in reduced sugar metabolism. The mycelia were cultured on PDB medium for 10 days, and then the three stresses were added to the medium for 24 h. We found that the PK activity was increased by 1.8-, 5.5- and 4.0 folds by addition of CdCl_2_, NaCl and H_2_O_2_, respectively (Fig. 6a). After 24 h of stress pretreatment, the mycelia were transferred into HRW for 48 h. Figure 6b shows that the PK activity was increased in the HRW-treated mycelia compared with the CK group. Besides, the expression of PK at the mRNA level was also induced by the HRW treatment.

### Gene expression analysis by qRT-PCR

**Fig. 7:**
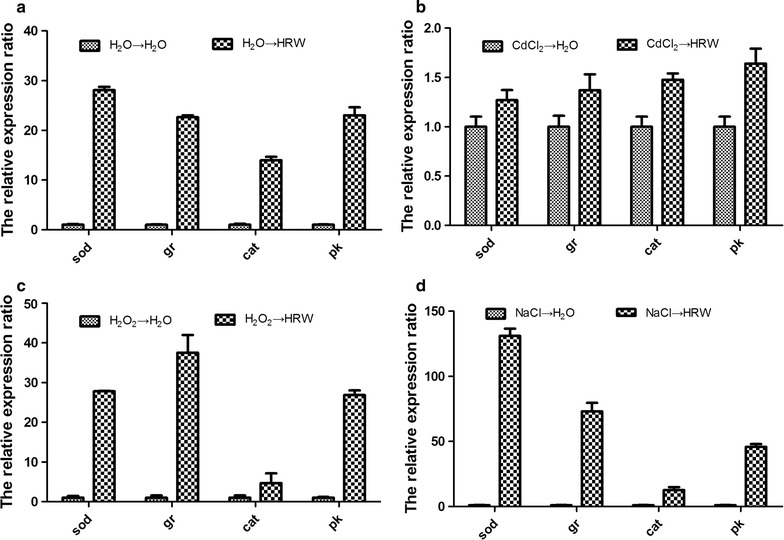
Effects of HRW treatment on gene expressions of sod (**a**), cat (**b**), gr (**c**) and noxR (**d**) in the mycelia of *Hypsizygus marmoreus* upon CdCl_2_, NaCl and H_2_O_2_ stresses. Expression levels of corresponding genes are presented relative to the control samples. Data are mean ± SE from three independent experiments

To confirm the above-mentioned findings, we examined the expressions of genes encoding SOD, CAT, GR and PK. The 18 s ribosomal RNA was used as a housekeeping gene, and the ddH_2_O-treated mycelia under the stressed conditions were used as the CK samples. Figure 7 reveals that the expressions of the above-mentioned four genes were all increased after the HRW treatment. In the CdCl_2_ pretreatment, the expressions of the four genes were induced by the HRW treatment at the lowest level. CAT most sensitively responded to the CdCl_2_ pretreatment. In the Na pretreatment, the expressions of the four genes were induced by the HRW treatment at the highest level. SOD most sensitively responded to the Na pretreatment, and its expression level was increased by 135 folds compared with the CK group. In the H_2_O_2_ pretreatment, GR most sensitively responded to the pretreatment, showing an increased expression of 37.5 folds.

## Discussion

*Hypsyzigus marmoreus* is one of commercial mushrooms. In 2012, the daily production of *H. marmoreus* was 167 tons in China (Zhang et al. 2016a) according to data from the China Edible Fungi Association, suggesting the rapidly increased demand for *H. marmoreus* and consumption of substrates. In edible mushrooms, they are usually grown on agricultural wastes which may contain toxic substances, such as heavy metals. These adverse factors may inhibit the mushroom growth and affect the fruit body production. In mushrooms, many previous studies have reported the effects of heavy metals and H_2_O_2_ on the growth of edible mushrooms (Hatvani and Mecs 2003; Zharare et al. 2010).

In this present study, we first assessed the effects of CdCl_2_, NaCl and H_2_O_2_ at different concentrations on the mycelial growth of *H. marmoreus*. CdCl_2_ (50 µM), NaCl (1.0%) and H_2_O_2_ (2 mM) could cause about 50% reduction in mycelial growth in solid medium. The mycelia became thinner compared with the control as the concentrations of CdCl_2_ and NaCl were increased. In contrast, H_2_O_2_ exhibited an insensitive inhibitory effect on mycelial growth compared with CdCl_2_ and NaCl, and the mycelial morphology under the H_2_O_2_ stress was similar with the control (Fig. 2a). These results were in accordance with the studies of Zhang et al. (2011) and Zharare et al. (2010), which showed that the mycelial growth and biomass of *H. marmoreus* are significantly decreased with addition of CdCl_2_, NaCl or H_2_O_2_, and CdCl_2_ had a greater harmful effect than NaCl and H_2_O_2_.

Besides, the levels of H_2_O_2_ (Fig. 5a, c) and O_2_^−^ (Fig. 5a) were also enhanced by these three pretreatments. Under most conditions, ROS (H_2_O_2_ and O_2_^−^) can be efficiently scavenged by antioxidants, such as SOD, CAT and GR (Xie et al. 2012; Zhang et al. 2016b). In our study, pretreatments of CdCl_2_, NaCl and H_2_O_2_ significantly decreased the CAT and GR activities in mycelia, whereas the SOD activity was induced by the three pretreatments, which were consistent with the results of gene expression (Fig. 7). In mushrooms, antioxidants exhibit different responses to different stresses (Jiang et al. 2015; Zhang et al. 2016a). These results suggested that the high level of H_2_O_2_ or O_2_^−^ in CdCl_2_, NaCl and H_2_O_2_-stressed mycelia was caused, at least in part, by an impaired detoxifying capacity of ROS. Similar with plants, some abiotic stresses, including heavy metals or salinity, can directly or indirectly cause damages to mushrooms by ROS production, which can induce lipid peroxidation and antioxidant responses in plants or mushrooms (Cao et al. 2012; Maria and Bebianno 2011). Our study also demonstrated that the pretreatments with CdCl_2_, NaCl and H_2_O_2_ enhanced MDA levels in mycelia of *H. marmoreus* (Fig. 5b), which is an index of lipid peroxidation and oxidative stress.

In plants, HRW has been found to enhance the antioxidant capacities of inducing plant tolerance to some stresses, such as salinity, CdCl_2_ and blue light-induced oxidative stress (Cui et al. 2013; Xie et al. 2012; Zhang et al. 2015c). In fungi, there is no report about the effects of HRW on the growth and antioxidant capacity. Our results demonstrated that HRW treatment ameliorated the adverse effects of CdCl_2_, NaCl and H_2_O_2_ in the mycelia of *H. marmoreus*. First, we found that the mycelia after the HRW treatment for 5 days were more quickly regenerated than the control group (Fig. 2a). Figure 2b and c reveal that the mycelial growth was enhanced and the mycelial biomass was increased after the HRW treatment. Besides, the CdCl_2_, NaCl and H_2_O_2_ accumulation in mycelia were attenuated after HRW treatment (Table 1). In plants, HRW can also enhance the growth response to different toxic factors, such as CdCl_2_ and NaCl (Cui et al. 2013; Xie et al. 2012; Zhang et al. 2015a). Our results indicated that HRW could effectively alleviate the growth inhibition and oxidative damage triggered by CdCl_2_, NaCl and H_2_O_2_ stresses in mycelia of *H. marmoreus*, which was in accordant with the previous study in plants.

Second, we found that HRW could enhance the antioxidant capacity of the mycelia in *H. marmoreus*. The HRW treatment led to a decrease in oxidative injuries caused by CdCl_2_, NaCl and H_2_O_2_, and the level of MDA was decreased (Fig. 5c). This finding was in good agreement with the reduced accumulation of ROS (H_2_O_2_ and O_2_^−^) and increased activities of antioxidants (SOD, CAT and GR). Besides, the HRW treatment also induced the expressions of antioxidants (SOD, CAT and GR) at the mRNA level. These results suggested that HRW might decrease lipid peroxidation in mycelia under these three stresses by activating antioxidants. In plants, such as rice (Xu et al. 2013), Arabidopsis (Xie et al. 2012) and Chinese cabbage (Wu et al. 2015a), HRW has the similar effects. One possible role of HRW might be that H_2_ can readily permeate the cell membrane, thereby increasing the gene expression of antioxidants (Cui et al. 2013), which is similar with our data. It has been observed in an in vitro experiment that HRW is able to directly quench H_2_O_2_, but not singlet oxygen radical (Xie et al. 2012). However, HRW could effectively scavenge H_2_O_2_ and O_2_^−^ in the mycelia of *H. marmoreus* (Fig. 5).

In addition, HRW treatment could also enhance the PK activity in mycelia of *H. marmoreus* (Fig. 6a), which is a critical enzyme in glycolytic pathway. This result was in a good agreement with the increased expression of PK at the mRNA level (Fig. 6b). It has long been recognized that energy metabolism is linked to the production of ROS, and critical enzymes allied to metabolic pathways can be affected by redox reactions (Quijano et al. 2016). Mitochondria are primary sites of intracellular formation and reaction of ROS during the glucose metabolism (Brookes et al. 2004; Vasquez-Vivar et al. 2000). *Lyngbya* sp. can produce H_2_ during this metabolism (Shi and Yu 2016). In rice, HRW can activate α/β-amylase activity, thus accelerating the formation of reducing sugar and total soluble sugar (Xu et al. 2013). These results suggested that HRW regulated the level of ROS to enhance the metabolic efficiency of sugar and provided more energy for mycelial growth.

In conclusion, we found that hydrogen-rich water (HRW) treatment could alleviate the toxicities of CdCl_2_, NaCl and H_2_O_2_, leading to improved mycelial growth and biomass. The HRW treatment decreased the levels of malondialdehyde (MDA) and ROS and significantly increased the activities of antioxidants (SOD, CAT and GR). These results suggested that HRW treatment might enhance the antioxidant abilities to induce the mycelial growth in *H. marmoreus*. Besides, pyruvate kinase was activated by HRW treatment, suggesting that HRW treatment also activated the glucose metabolism. These results suggested that the usage of HRW could be an effective approach for contaminant detoxification in *H. marmoreus*.

## Additional file

**Additional file 1: Table S1.** Primer sets used for quantitative real-time PCR.

## Abbreviations

HRW: hydrogen-rich water
ROS: reactive oxygen species
MDA: malondialdehyde
SOD: superoxide dismutase
CAT: catalase
GPX: glutathione peroxidase
POD: guaiacol peroxidase
PK: pyruvate kinase
DAB: 3, 3, 9-diaminobenzidine
NBT: nitroblue tetrazolium
