# Supplementary material for: Hydrogen-rich water alleviates the toxicities of different stresses to mycelial growth in Hypsizygus marmoreus
Source: AMB Express. 2017 May 30;7:107. doi: 10.1186/s13568-017-0406-1 (PMC5449350; doi:10.1186/s13568-017-0406-1)
Supplement: Supplementary file 1 — Additional file 1: Table S1. Primer sets used for quantitative real-time PCR. [file 13568_2017_406_MOESM1_ESM.docx]

**Table S1.** Primer sets used for quantitative real-time PCR.

| Unigene name | Sequence (5^’^-3^’^) | Accession No. |
| --- | --- | --- |
| CAT-F  CAT-R | CAACCGTCCGTTTCTCCACT  CCAATCCCAATTACCTTCCTC | GBCL01039326 |
| GR-F  GR-R | TAAATGATTTGGTGGAGGC  CATCGGTGAAACTGAGGC | GBCL01010302 |
| SOD-F  SOD-R | CGCTAATGGTGAAGGCGGAAAG  TTCGGAGTGGTCACAATCTCAAGC | GBCL01008983 |
| PK-F  PK-R | GTTTAATGGTTGCTCGTGGTG  CCATCGTAGATTGCGTTTGC | GBCL01019135 |
| 18s-F  18s-R | GAGGGACCTGAGAAACG  ATAAGACCCGAAAGAGCC | KC510993 |
